# Comparative evaluation of additively manufactured PLA models as cost-effective, non-inferior alternatives to composite bones for pull-out testing

**DOI:** 10.1007/s00402-026-06278-4

**Published:** 2026-03-28

**Authors:** Utku Demirtaş, Osman İyibilgin, Levent Bayam, Erdinç Genç

**Affiliations:** 1 Department of Orthopaedics and Traumatology, Sultan 1. Murat State Hospital, Edirne, Turkey; 2 Mechanical Engineering Department, Sakarya University, Sakarya, Turkey; 3 Biomaterials, Energy, Photocatalysis, Enzyme Technology, Nano and Advanced Materials, Additive Manufacturing, Environmental Applications and Sustainability Research and Development Group, Sakarya University, Sakarya, Turkey; 4 Department of Orthopaedics and Traumatology, Istanbul Medipol University, Istanbul, Turkey

**Keywords:** Additive manufacturing, Polylactic acid (PLA), Composite bone model, 3D-printed bone model, Pull-out strength, Mechanical testing

## Abstract

**Introduction:**

The purpose of this study was to perform biomechanical pull-out comparisons between composite bone blocks and additively manufactured bone models using locked plate and screw systems. The hypothesis posited that the mean pull-out force of the 3D-printed models would demonstrate non-inferior pull-out strength compared to standard composite bones within a clinically acceptable 15% margin. A standardized pull-out mechanism was developed to evaluate its biomechanical reliability and its potential as a customizable alternative.

**Materials and methods:**

The study compared three groups: standard composite (Sawbones) blocks and two types of additively manufactured polylactic acid (PLA) blocks (grid and gyroid infill pattern), each measuring the same dimensions with 30% infill and mimicking a Sawbones block with 2 mm cortical thickness. A 6-hole LC-DCP titanium plate was fixed onto bone block models with dimensions of 29 × 170 × 42 mm using four screws, each measuring 36 mm in length, which were inserted perpendicularly and tightened to the same torque levels. Steel cables placed under the plate were connected to a mechanical testing setup, and pull-out tests were conducted to evaluate fixation strength. Seven replicate specimens were fabricated to ensure repeatability and reliability of the results.

**Results:**

Mean pull-out forces were 1784.4 ± 79.9 N for Sawbones, 1698.5 ± 134.3 N for Grid PLA (*n* = 7), and 1908.7 ± 335.2 N for Gyroid PLA (*n* = 7). Statistical analysis using a one-sample t-test showed that both 3D-printed models were non-inferior to the standard Sawbones model within a clinical acceptance margin of 15% (*p* < 0.05).

**Conclusions:**

Additively manufactured models exhibit mechanical pull-out performance that is statistically non-inferior to traditional composite bone block models. These findings confirm that 3D-printed PLA-based grid and gyroid structures serve as reliable, cost-effective alternatives for orthopaedic mechanical research without compromising mechanical integrity.

## Introduction

Biomechanical analyses are widely used in orthopaedic research applications. By forecasting how implants behave under stress in bone tissue, they contribute to implant design, arthroplasty applications, and the efficacy of surgical treatment. Cadaveric bones have been utilized in investigations for an extended period; although they yield results that approximate the behaviour of living bone under physiological conditions, they exhibit several significant drawbacks. The most notable disadvantages include cultural limitations, degradation over time, ethical dilemmas, high costs, potential toxicity, demanding storage requirements, resource availability from suppliers, and regulatory shipping protocols [1, 2]. Furthermore, cadaveric specimens are disproportionately representative of geriatric individuals, and their bone quality may not accurately depict the general orthopaedic patient demographic [1, 3].

Composite bone models represent living bone from a biomechanical perspective and consisted of a rigid polyurethane foam core surrounded by an epoxy-reinforced woven glass shell. Despite the fact that first-generation models did not yield fully biomechanically accurate results, fourth-generation composite bone models were created using a pressure injection technique with short glass fiber reinforced (SGFR) epoxy on a polyurethane foam core to form cortical bone [1]. Consequently, this created an ideal model for reloading applications and biomechanical testing under physiological conditions [3]. Despite their cost-effectiveness relative to cadaver samples due to their high mechanical consistency, low variability, and usability, these composite bone models have various limitations that reduce their accuracy in simulating human bone [3]. When utilized to simulate specific fracture types, these models generate bone-implant structures that exhibit unrealistic stability in comparison to actual cadaver bone. They also result in atypical fracture patterns and differ in their anthropometric and mechanical properties [4, 5]. Despite the reduced expense of the unit cost in comparison to cadaver samples, the overall cost exhibits a notable increase when substantial sample series are conducted, such as multiple loading tests and screw pull-out tests.

The various limitations of composite bones previously mentioned offer opportunities to overcome these constraints through advances in 3D printing technology. Extrusion-based methods are additively manufactured (AM) processes in which the material is deposited through a nozzle, layer by layer, to build the desired three-dimensional structure. Among these technologies, Fused Filament Fabrication (FFF) has emerged as a simple, versatile, and cost-effective approach capable of multi-material printing. However, this technique is limited by relatively low resolution and often requires post-processing to improve surface quality and dimensional accuracy [6]. A review of literature regarding 3D printing outcomes reveals a wide range of applications in diverse surgical domains. Orthopaedic surgery dominates the field, accounting for 45.18% of the total publications, with printing techniques being most frequently employed for surgical guides and models utilized for surgical planning [7]. However, with the advancement of technology, it has also begun to be used extensively in biomechanical studies. While these studies validate the use of AM models, research standardizing these studies is limited. Therefore, creating a verified standard protocol that utilizes 3D printing technology would both lower expenses and make biomechanical research more accessible, enabling facilities with constrained budgets to perform rigorous implant evaluations.

The purpose of this study was to perform biomechanical comparisons between composite bone blocks and additively manufactured bone blocks produced using FFF using locked plate and screw systems. The pull-out strength functions as a critical parameter for evaluating the integrity of the screw-bone interface and predicting possible fixation failures in clinical scenarios [8]. The hypothesis posited that the mean pull-out strength of the 3D-printed blocks was equal to or lower than this threshold. A standardized pull-out mechanism was developed to evaluate its biomechanical reliability and its potential as a customizable alternative.

## Materials and methods

### Fabrication of test specimens using a 3D printer

**Fig. 1:**
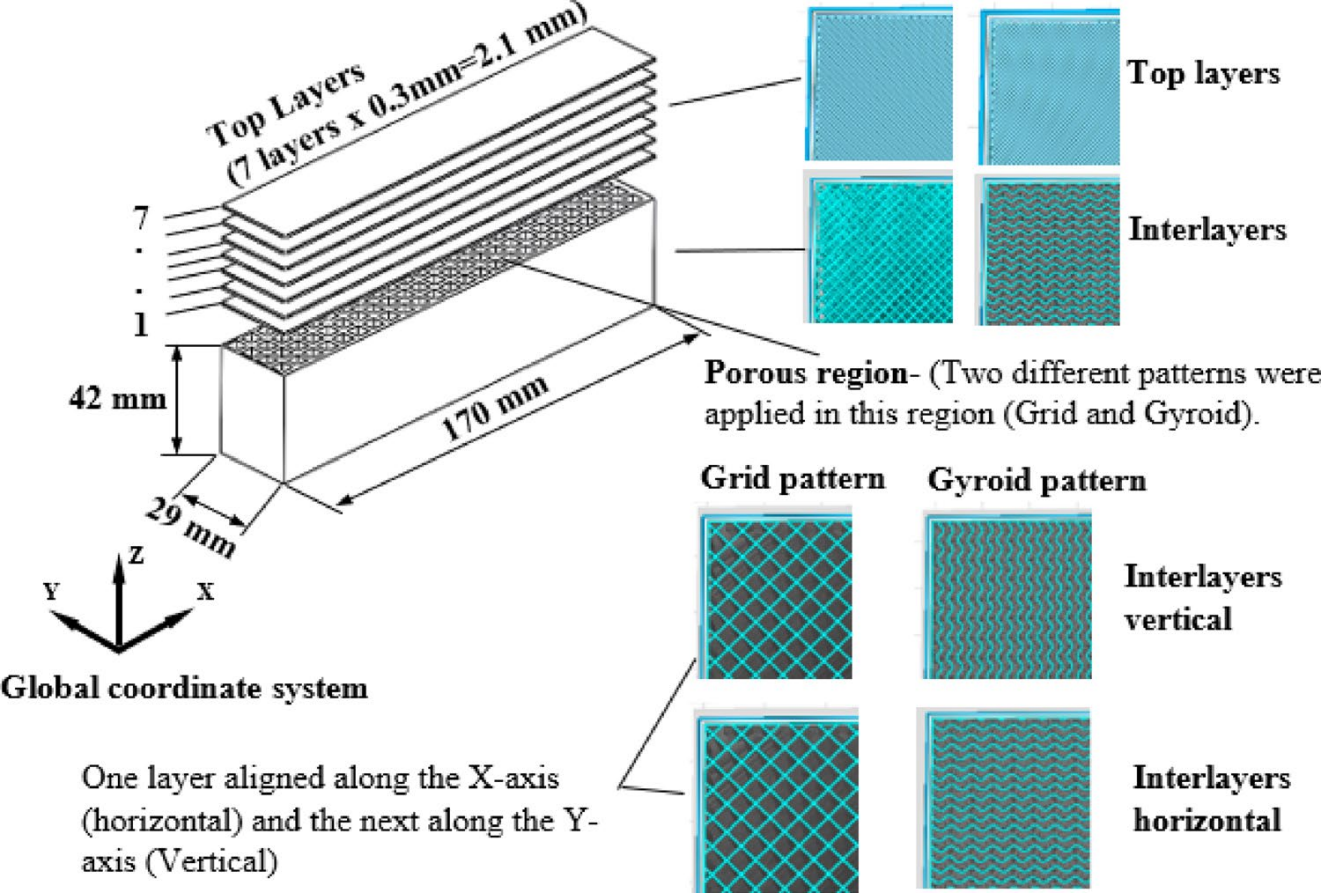
FFF-printed test blocks used for mechanical evaluation. All specimens were fabricated using a layer-by-layer deposition process along the Z-axis (build direction). Within the XY plane, alternating raster orientations were applied, such that one layer was deposited with filament strands aligned along the X-axis and the subsequent layer along the Y-axis (0°/90° configuration). At 30% infill density, this alternating orthogonal deposition generated the internal grid architecture

All specimens were produced using a 3D printer with polylactic acid (PLA) filament of 1.75 mm diameter, using FFF technology with 30% infill density. These specimens were modelled in SolidWorks as rectangular shapes with dimensions of 29 × 42 × 170 mm. The build direction corresponded to the Z-axis. Within the XY plane, a 0°/90° alternating raster orientation was applied, meaning that successive layers were deposited orthogonally (one layer aligned along the X-axis and the next along the Y-axis). This configuration generated a cross-hatched internal structure in the grid pattern and ensured mechanical anisotropy consistent with typical FFF fabrication [9] (Fig. 1).

The width of the AM models was designed as 29 mm, compared to the 60 mm width of the standard composite Sawbones blocks. This dimension was selected to optimize AM efficiency while ensuring biomechanical validity. Screw pull-out failure is primarily governed by the shear strength of the material at the thread interface rather than the global bending stiffness or total volume of the block [8, 10]. Furthermore, in accordance with ASTM F543-17 guidelines for testing medical bone screws, the test block dimensions must be sufficient to prevent edge effects [11]. Given the 3.5 mm screw diameter, the 29 mm block width provided approximately 12.75 mm of surrounding peri-implant material on each side. This margin was sufficient to fully contain the embedded thread length and prevent lateral wall failure during testing, ensuring that the local mechanical conditions and failure modes remained directly comparable to those of the wider standard composite blocks. In the tests, specimens made from PLA were compared. Application of AM facilitates production of models with unique bone geometries, material combinations, and infill patterns, thereby enabling cost-effective manufacturing processes. Consequently, two PLA blocks were fabricated, each exhibiting a distinct internal pattern (gyroid and grid pattern) and presenting a comparable appearance and weight. The fourth-generation composite bone analogue used in this study was a standardized solid rigid polyurethane foam block laminated with a 2 mm short glass fiber–reinforced epoxy cortical layer (Sawbones, Pacific Research Laboratories, Inc., USA). Sawbones was used as a fixed standard reference model. The cancellous component consists of closed-cell polyurethane foam with a density of 20 pcf (0.32 g/cm³) conforming to ASTM F-1839-08 standards. This material ensures uniform structural consistency (±10% density variation) and provides mechanical properties within the range of human cancellous bone and is widely recognized as the standard reference medium for evaluating screw pull-out strength and fixation stability [12]. The cortical layer is composed of short glass fiber–filled epoxy, designed to simulate the stiffness and load-bearing behaviour of human cortical bone. The block dimensions were 60 × 42 × 170 mm.

To simulate the structure of Sawbones in the 3D-printed specimens, seven solid layers were printed, resulting in a total thickness of 2.1 mm. This thickness corresponds to the cortical bone thickness found in Sawbones models. All test specimens were produced using a ZAXE-brand ZAXE^®^ FDM 3D printer (Zaxe Inc., Istanbul, Türkiye). 3D printer with a 0.4 mm diameter nozzle, 0.5 mm line width, 0.3 mm layer height, 30% infill density with 50 mm/s print speed, 205 °C nozzle tempature, 60 °C bed temperature, and a filament flow rate/extrusion multiplier of 100% (1.00).

Regarding the internal architecture, for the grid pattern, the in-plane center-to-center spacing between adjacent filament strands was approximately 1.6–1.7 mm, reflecting the effective spacing generated by the slicer algorithm. This 1.6–1.7 mm macro-spacing closely aligns with the upper spectrum of natural trabecular spacing (Tb.Sp) found in human cancellous bone. According to morphometric studies, Tb.Sp typically averages between 0.64 and 0.75 mm across different anatomical sites, and ranges from ~0.51 mm in dense femoral heads up to 1.5 mm in less dense conditions [13, 14]. As established by Wu et al., utilizing standard 0.4 mm extrusion nozzles limits exact microscopic replication; however, employing this geometrically scaled-up macro-porosity effectively mimics the bulk mechanical properties of cancellous bone [14]. For the gyroid pattern, the internal structure forms a triply periodic minimal surface (TPMS) geometry rather than discrete parallel filaments; thus, direct strand-to-strand spacing cannot be defined. However, its effective structural cell size was correspondingly governed by the 30% infill density to maintain a comparable bulk material fraction. Furthermore, broader biomechanical validations using various 3D printing modalities have consistently demonstrated that AM porous structures accurately reproduce the mechanical stiffness and strength of real bone under loading [15, 16]. Therefore, our specific architectural scaling allows the printed models to adequately simulate the macroscopic mechanical resistance during local screw thread engagement.

For the internal architectures, the grid pattern was fabricated by alternating the FFF extrusion toolpaths (0° and 90° raster angles) layer-by-layer. While the grid inherently possesses a 90-degree rotational symmetry macroscopically, this alternating orthogonal deposition creates intersecting nodes that thermally fuse, effectively enhancing interlayer adhesion and shear strength by preventing the structure from splitting along parallel extrusion lines. In contrast, the gyroid pattern consists of a continuous TPMS, relying on its complex, non-linear geometric cross-sections across the printing layers to distribute mechanical loads.

### Test specimen preparation

In this study, two types of bone analog models were fabricated to investigate the mechanical performance differences in pull-out tests using an LC-DCP (limited contact-dynamic compression plate) construct. The specimens were produced using additive manufacturing with two distinct internal infill patterns: grid and gyroid. Both infill types were manufactured with a 30% infill density, which was chosen to simulate the mechanical properties of cancellous bone while maintaining consistency across all samples [17].

Each specimen was designed in a standard geometric shape appropriate for the pull-out test setup, ensuring compatibility with the LC-DCP construct. A total of fourteen specimens were prepared, with seven samples produced for each infill type to allow for reliable non-inferiority testing.

### Testing equipment and setup

Mechanical testing was performed using a Dartec servo-hydraulic universal testing machine (Dartec, UK), which provides precise load control and displacement measurement capabilities. The pull-out test setup was designed to simulate the mechanical loading conditions experienced by locking screws in healthy bone.

A 6-hole LC-DCP humeral locking plate (TST, Istanbul, Türkiye) was used in this study. The plate is manufactured from medical-grade titanium alloy (Ti-6Al-4 V ELI). The plate dimensions were 3.5 mm thickness, 12 mm width, and 115 mm length. A total of four screws, each measuring 36 mm in length, were used in the experiment. Of this length, 28 mm was embedded within the test material, while the remaining 8 mm was left exposed above the surface to allow for the application of pull-out force. Locking cortical screws compatible with the plate system (3.5 mm diameter, titanium) were inserted along the Z-axis (parallel to the build direction). Therefore, during pull-out testing, the screw threads primarily engaged the interlayer interfaces, and mechanical resistance was largely influenced by interlayer bonding strength rather than continuous in-plane filament strands. Screws tightened perpendicular to the plate and tightened with standardized torque. To ensure consistent fixation quality and prevent thread stripping, all screws were inserted using a calibrated torque-limiting screwdriver standardized at 1.5 Nm, in accordance with the manufacturer’s specifications for the 3.5 mm locking system.

### Pull-out test

**Fig. 2:**
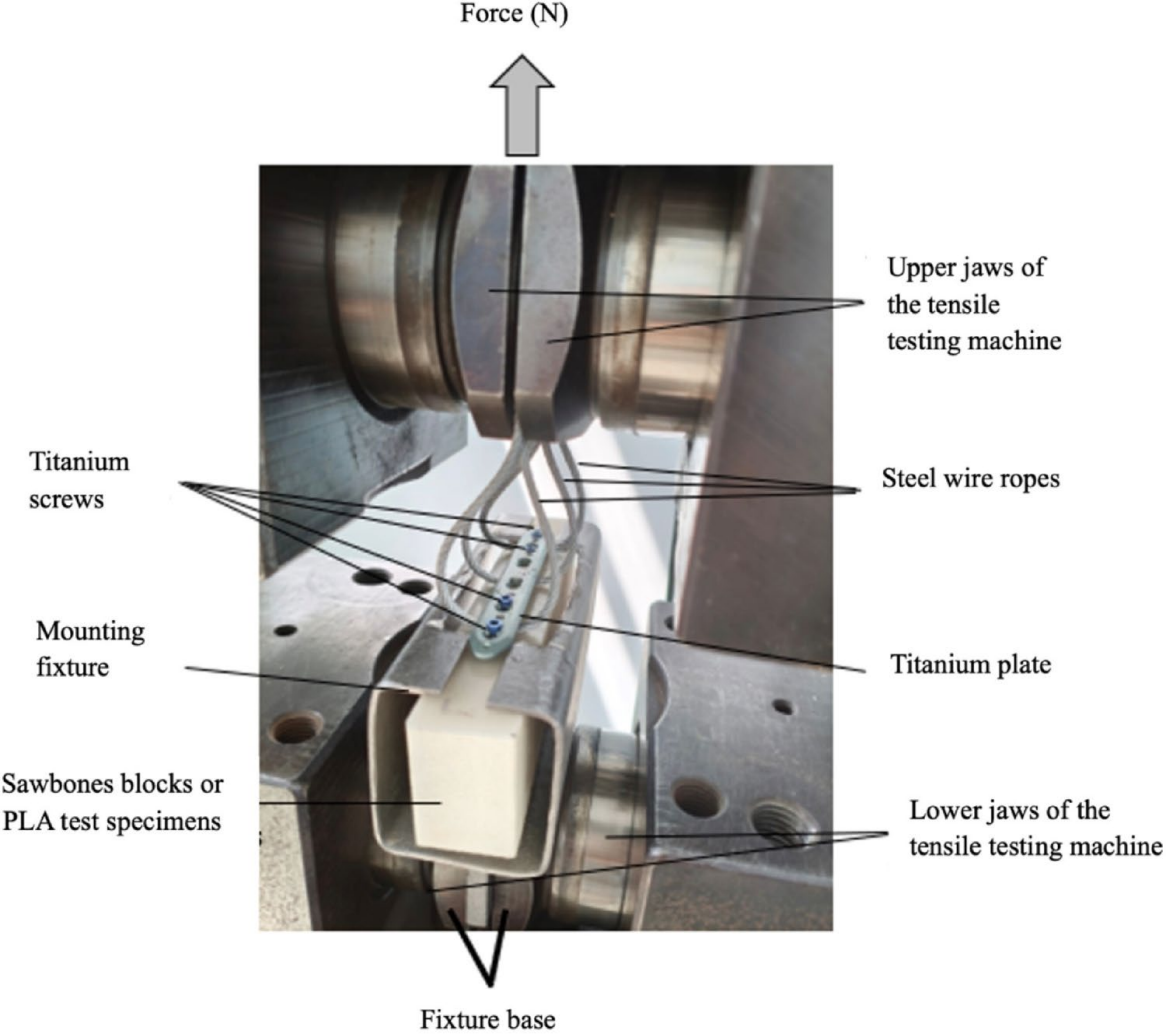
Experimental apparatus

To perform the pull-out test, a custom apparatus was designed to enable the removal of the plate. This apparatus allowed performing pull-out tests of cortical screws. The experimental setup and equipment used in the study are shown in Fig. 2.

In this experimental study, four locking cortical screws were employed, and their extraction was facilitated using three steel cables, as depicted in Fig. 2. The pull-out force was applied via high- strength, multi-strand steel wire ropes (3 mm) attached to the locking plate at equidistant intervals along its longitudinal axis. This configuration connected the plate construct to the upper grip of the testing machine, ensuring self-alignment and a uniform distribution of the pull-out force across the entire plate-screw construct. The pull-out tests were conducted using a Dartec universal testing machine under displacement control, with a constant crosshead speed of 5 mm/min. For each test condition, seven replicate specimens were fabricated to ensure repeatability and reliability of the results. The mean values of the measured pull-out forces were calculated and used for subsequent analysis.

### Statistical analysis

Statistical analyses were performed using JASP software (Version 0.95.4, University of Amsterdam). Descriptive statistics, including mean and standard deviation (SD), were calculated for pull-out forces. Due to the limited sample size of the control group (Sawbones, *n* = 2), a one-sample t-test approach was utilized to evaluate the non-inferiority of the additively manufactured (AM) models against a fixed reference standard. The mean pull-out force of the Sawbones model (1784.4 N) was established as the reference value. A clinically acceptable non-inferiority margin was defined as 15% of the reference mean (M = 267.7 N), resulting in a non-inferiority threshold of 1516.7 N. A 15% non-inferiority margin was selected based on the coefficient of variation typically observed in biomechanical testing of AM structures and composite models [1]. Given that the standard deviation in the gyroid group represented approximately 17.5% of the mean pull-out force (Table 1), a 15% margin provides a rigorous yet clinically appropriate threshold for establishing equivalence. The normality of the data distribution was verified using the Shapiro-Wilk test (*p* > 0.05). A p-value of < 0.05 was considered statistically significant, indicating non-inferiority. Data visualization and the generation of figures were performed using custom scripts written in the Python programming language (utilizing the Matplotlib library) to ensure high-resolution and precise data representation.

## Results

**Fig. 3:**
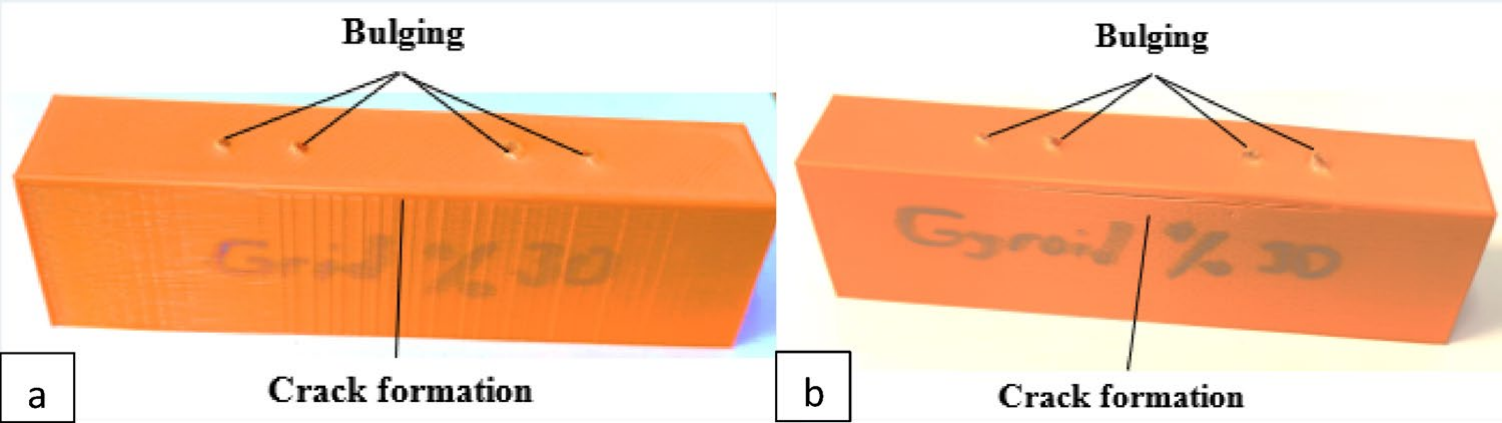
Screw hole morphology after pull-out testing. The macroscopic photographs show local deformation around the screw threads, including the degree of filament compression, structural collapse and presence or absence of interlayer separation for the **a** grid and **b** gyroid pattern

Microstructural failure patterns differed slightly due to architectural differences; the macroscopic pull-out behavior and failure morphology were comparable (Fig. 3).

### Pull-out strength

**Fig. 4:**
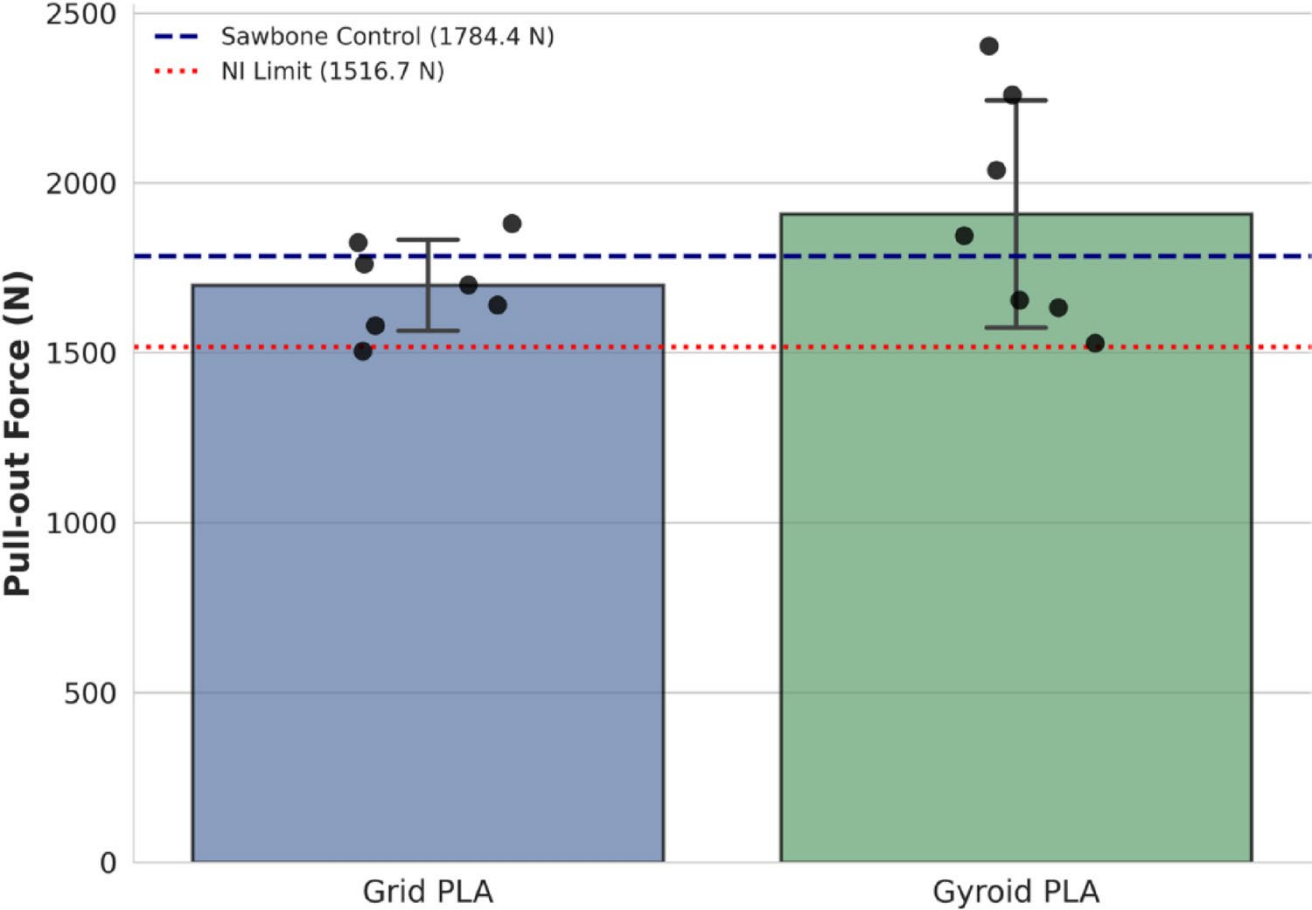
Comparison of pull-out forces between AM models (grid and gyroid) and the Sawbones reference. The red dashed line represents the non-inferiority (NI) limit. Individual data points are superimposed to show distribution. The blue dashed line represents the Sawbones mean, and error bars indicate standard deviation

Figure 4 presents the pull-out force values obtained from specimens made of two different materials and printed with two different internal patterns.

**Fig. 5:**
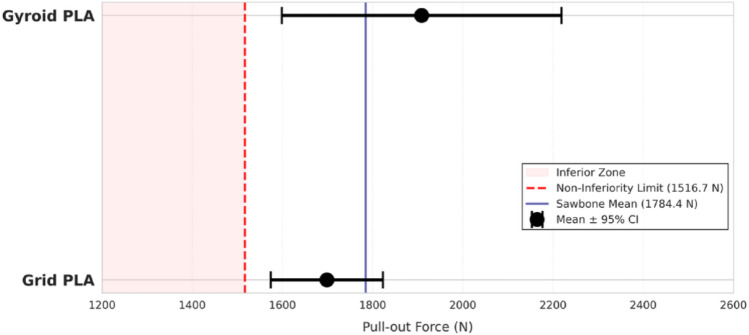
Non-inferiority analysis showing mean pull-out forces and 95% confidence intervals (CI). The red vertical dashed line indicates the non-inferiority limit (1516.7 N), calculated as a 15% margin from the Sawbones mean. Since the 95% CIs for both groups lie entirely to the right of the red line, non-inferiority is established

The results indicate that the infill pattern and material type both influence the mechanical strength in pull-out testing. Figure 5 represents non- inferiority analyses obtained from the pull-out tests for Sawbones and PLA specimens with grid and gyroid infill patterns.

The descriptive statistics for the pull-out forces are presented in Table 1 and in Fig. 4. The Sawbones group exhibited a mean pull-out force of 1784.4 N. The Grid and Gyroid infill patterns demonstrated mean forces of 1698.5 ± 134.3 N and 1908.7 ± 335.2 N, respectively.

**Table 1:** Descriptive statistics of pull-out forces

Group	*n*	Mean (*N*)	SD (*N*)	Min (*N*)	Max (*N*)	CV (%)	95% CI
Sawbones	2	1784.4	79.9	1727.9	1840.9	4.5%	-^a^
Grid PLA	7	1698.5	134.3	1504.6	1880.6	7.9%	1573.8–1822.2
Gyroid PLA	7	1908.7	335.2	1527.8	2403.4	17.6%	1598.9- 2219.1

^a^ 95% CI was not calculated for the Sawbones group due to the sample size, which precludes reliable interval estimation

*n* number of specimens; *N* Newton; *SD* Standard deviation; *Min* Minimum; *Max* Maximum; *CV* Coefficient of variation; *CI* Confidence interval

The Shapiro-Wilk test confirmed the normal distribution of the data for both AM groups (*p* > 0.05). The one-sample t-test for non-inferiority revealed that the pull-out strength of the grid PLA model was significantly higher than the non-inferiority threshold of 1516.7 N (*p* < 0.006). Similarly, the gyroid PLA model also demonstrated non-inferiority, significantly exceeding the threshold (*p* = 0.011). These results statistically confirm that the mechanical performance of both 3D-printed infill patterns is within the accepTable 15% range of the standard composite bone models in Table 2.

**Table 2:** Non-inferiority analysis results against the threshold of 1516.7 N

Group	Mean ± SD (*N*)	95% CI lower bound (*N*)	Safety margin (*N*)^a^	*p*-value	Result
Grid PLA	1698.5 ± 134.3	1574.1	+ 181.8	0.006	Non-Inferior
Gyroid PLA	1908.7 ± 335.2	1598.7	+ 392.0	0.011	Non-Inferior

^a^ Safety Margin represents the difference between the mean pull-out force and the non-inferiority threshold

*SD* Standard deviation; *N* Newton; *CI* Confidence interval

The minor discrepancies observed may be attributed to variations in the rotational alignment of the screw threads relative to the printed layers during fixation, rather than intrinsic material inconsistencies. The discrepancies seen in the error bars in Fig. 3 could be due to this reason. Although Sawbones exhibits higher displacement values, the displacement values obtained from PLA specimens are consistent with those of bone structures [18].

## Discussion

This is the first study to compare composite and AM bone blocks directly using a pull-out test setup. The primary finding of this study is that PLA-based AM bone models, specifically with grid and gyroid infill patterns, are mechanically non-inferior to fourth-generation composite Sawbones blocks in screw pull-out tests. By applying a statistical non-inferiority analysis, we demonstrated that the slight reduction in mean strength observed in the grid pattern was not statistically inferior to the clinical threshold. Furthermore, the gyroid pattern not only met the non-inferiority criteria but also exhibited mean pull-out forces numerically higher than the control group, suggesting a robust mechanical structure potentially superior for high-load scenarios. These findings suggest that 3D-printed AM PLA bone blocks can serve as a reliable and cost-effective alternative to commercially available composite bone models for biomechanical testing involving locking plate constructs.

Our results align with and expand upon previous research validating AM bone models with adjustable mechanical properties that closely mimic natural bone and the strength of trabecular bone [19, 20].

Plate screw constructs in the orthopaedic practice are usually configured with six screws, tree screws per fragment. This configuration has been widely adopted, and it offers an optimal balance of construct stability. From a biomechanical perspective, without resulting in an unwarranted increase in stiffness, the usage of three screws per segment is typically adequate to ensure sufficient load distribution and to minimize stress concentration along the plate. However, composite and AM blocks exhibited healthy bone density, resulting in higher inherent pull-out resistance of the screws. Initially, preliminary testing with a six-screw configuration revealed excessive construct rigidity, which inhibited the displacement necessary to evaluate the specific plate-bone block interference. Consequently, the configuration was optimized to four screws (two per fragment) to facilitate measurable pull-out failure mechanics while preserving clinically relevant construct behaviour. This configuration was implemented to ensure the structural integrity of the construct was maintained while enabling the assessment of the screw-bone interface performance under realistic stress levels. As indicated by the extant literature, analogous approaches have been described, suggesting that a reduced but strategically placed number of screws can maintain sufficient stability without compromising construct behaviour or load transfer characteristics [21, 22].

While various filament materials exist, PLA filament is selected due to its predictable mechanical behaviour, cost-effectiveness, compatibility with various printer brands, minimal deformation, and resistance to warping. This facilitates the control of printing parameters and ensures dimensional repeatability and geometric accuracy between samples [9, 23]. The gyroid structure is a TPMS geometry that emulates the isotropic stiffness properties of trabecular bone structure [24]. The gyroid pattern facilitates greater resistance against screw pull-out. While this pattern does not contribute to cortical bone strength and structure, it has been shown to mimic the complex structure of bone trabeculae [25, 26]. The grid infill pattern arrangement facilitates predictable load transfer and stiffness along the primary printing axes, thereby offering advantages for comparative mechanical testing and modelling [27]. However, this configuration may result in the development of localized stress concentrations, which can potentially lead to premature micro failure during the pull-out mechanism test [25]. The orthogonal insertion direction of the screws relative to the printing plane (parallel to the Z-axis) significantly influenced the mechanical outcomes. Because the axial pull-out forces directly stressed the weak interlayer bonds, the grid pattern’s planar structure made it consistently prone to interlayer separation. As seen in Table 1, this predictable failure mode resulted in a lower pull-out strength with CV. In contrast, the continuous TPMS geometry of the gyroid pattern resisted catastrophic straight-plane splitting, contributing to its higher average pull-out resistance. However, the exact local density of the curving gyroid architecture varies depending on the screw’s precise insertion point. This variable thread-to-material engagement explains the higher standard deviation and CV observed in the gyroid specimens compared to the uniform grid.

Infill density is recognized as the primary determinant of mechanical properties in FFF-printed PLA parts, with literature consistently demonstrating a direct positive correlation between density and both tensile and compressive strength [1, 4]. A recent study investigated the relationship between Young’s modulus and gyroid infill patterns at various densities [17]. Based on these findings, an infill ratio of 30% was selected to stimulate the mechanical stiffness of human cancellous bone, rather than to match the absolute weight of the larger composite models. This ratio provides an optimal balance between structural integrity and material efficiency.

Earlier research has confirmed that bone models produced through AM accurately reproduce the topographic characteristics and mechanical behaviour of human bone tissue, frequently exceeding composite bone models in both biological authenticity and dimensional accuracy [14, 28–33]. More recent comparative studies have additionally shown that although commercially available composite bone models may display greater stiffness compared to cadaver specimens, the mechanical properties of 3D-printed PLA models exhibit closer resemblance to natural bone, with fracture behaviours that align with clinical observations in patients [17, 28].

3D-printed bone analogues offer numerous advantages beyond mechanical properties to those of composite bone analogues. During the design and production stages of AM, researchers have the ability to modify bone geometry, infill density, and material properties. Thereby, these features enable the testing of specific anatomical conditions, such as osteoporotic bone or localized bone defects. In the translational research pipeline, the high cost of fourth-generation composite bones often limits preliminary mechanical evaluations. The utilization of AM technology addresses this barrier by offering a substantial reduction in production costs. When evaluating the economic viability of these models, stating a simple ratio based solely on raw material prices overlooks essential operational overheads. However, when a comprehensive cost calculation is applied, accounting for PLA consumption, electricity, machine depreciation over the print cycle, and minor material waste, the relative cost-effectiveness of AM remains substantial. If the fully accounted in-house production cost of a single AM test specimen is defined as one base unit (1x), relying on high-fidelity fourth-generation commercial composite blocks introduces a significantly higher financial and logistical burden. Particularly for international institutions, international shipping and customs duties drastically inflate the procurement cost. Even when a commercial block is strategically sectioned into multiple testing specimens, the comprehensive cost per test unit remains over 6 times higher (> 6x) than the fully accounted AM equivalent.

More critically, the true economic advantage of AM becomes even more evident at the macro-scale of biomechanical research, where multiple replicates (e.g., *n* ≥ 5–7) are required for statistical power. Relying on commercial composite blocks necessitates continuous, scaling procurement and international shipping costs for every new test variable. In contrast, a 3D printer represents a one-time infrastructural investment, effectively reducing the recurring economic burden strictly to inexpensive local consumables. By eliminating the financial barriers of international procurement and enabling rapid, software-based customization of internal architectures, 3D printing serves as a highly sustainable platform. This democratizes biomechanical research, empowering institutions with limited resources to conduct high-volume, iterative orthopedic studies. Beyond this long-term cost-effectiveness, AM technology provides an unprecedented logistical benefit: the ability to seamlessly customize internal architectures, structural densities, and cortical dimensions via software. This highly accessible customizability establishes 3D printing as a versatile, sustainable platform for iterative biomechanical testing, eliminating the financial and temporal constraints of continuously procuring diverse standardized models.

The AM models validated in this study offer a robust platform for rapid prototyping and cost-efficient preliminary failure assessment, allowing ineffective designs to be identified early in the development cycle. While the grid pattern provides a rapid and sufficient baseline for initial testing, the gyroid pattern offers superior structural integrity for high-load scenarios, allowing researchers to choose the optimal balance between cost and performance.

Transitioning from commercial composite bones to in-house AM models introduces risks of structural variability and biomechanical misinterpretations if print parameters are poorly designed. To prevent the misuse of AM models, future studies must ensure rigorous standardization, conduct routine batch validation (e.g., basic mechanical testing), and utilize reverse engineering or advanced metrology for quality control [34]. Researchers must carefully define the structural scope of their models and avoid extrapolating findings beyond validated limits. However, relying exclusively on rigidly standardized, fixed-parameter commercial models carries its own biomechanical risks. Recent literature demonstrates that 4th-generation commercial synthetic bones can be overly rigid compared to actual elderly cadaveric bones, whereas appropriately designed AM surrogates more accurately mimic true cadaveric failure mechanisms [28]. Therefore, when parameters are transparently reported and properly validated, AM technology not only serves as a reliable alternative to industrial synthetic bones but provides a crucial, customizable platform to simulate specific clinical conditions, such as osteoporotic bone.

However, this study has several limitations. Proficiency in the CAD software utilized in the production process is a prerequisite. The quality of printed documents and the calibration of printers may vary among users and be influenced by workflow standardization. The cost of 3D printers, the variability of filament, and hardware maintenance, particularly the cost of nozzle replacement and nozzle wear, can lead to inconsistencies between the models produced. The fabrication of a single model requires more time compared to the production of composite bone models. This discrepancy can impose limitations on the efficiency of time management in biomechanical studies involving extensive sample series. Although the sample size of the control group was limited (*n* = 2), the high manufacturing consistency and low reported variability of fourth-generation composite bones justified their use as a fixed reference baseline [1]. In contrast, a larger sample size (*n* = 7) was employed for the AM groups to sufficiently capture any potential inter-specimen variability arising from the printing process. Furthermore, all bone analogues produced by AM were fabricated using PLA filament, with a single infill density (30%), and evaluated using only a unidirectional pull-out mechanism. Consequently, the present results may not accurately mirror the intricate multiaxial stresses present.

This study has established a foundation for the utilization of bone analogues produced with AM in biomechanical testing. Future studies could include other filaments, such as PETG, ABS, or PEEK, and systematically evaluate different filament assessments and different infill percentages to generate structures analogous to real bone tissue. The long-term durability and fatigue resistance of the material can be evaluated by application of cyclic or multi-axial loading conditions. Additionally, validating these experimental findings with finite element analysis simulations would provide a comprehensive understanding of stress distribution within the printed infill patterns.

### Clinical relevance

This study shows reliability and a cost-effective and accessible 3D-printing protocol for biomechanical testing, establishing non-inferiority to standard composite bones. By significantly reducing long-term operational costs and eliminating the logistical constraints of continuously procuring fixed-parameter commercial models, this method enables high-volume, iterative evaluations of orthopaedic implants. Ultimately, this approach facilitates democratized biomechanical research capabilities, empowering institutions with limited resources to conduct rigorous studies and accurately simulate diverse clinical conditions.

## Conclusions

The study demonstrated that both grid and gyroid infill patterns in AM PLA bone blocks exhibit pull-out strengths that are statistically non-inferior to standardized composite bone blocks within a clinically acceptable 15% margin. The findings suggest a tiered approach for translational research: the grid pattern offers a rapid, resource-efficient baseline for initial screenings, while the gyroid pattern provides superior structural integrity for high-fidelity mechanical evaluations. Consequently, 3D-printed PLA models serve as a scientifically robust, customizable testing platform, accelerating preclinical orthopaedic research while reducing reliance on expensive composite models.

## Data Availability

The datasets generated and analyzed during the current study are available from the corresponding author on reasonable request.
